# Patient awareness and attitudes towards minimally invasive surgery

**DOI:** 10.6026/9732063002001641

**Published:** 2024-11-05

**Authors:** Tauseef Kibria, Soofia Firdaus, Sushant Kumar Sharma, Mahima Srivastava, Tushar Saini, Md Ashraf Ali, Sadaf Kibria

**Affiliations:** 1 Department of General Surgery, Sri Krishna Medical College & Hospital, Muzaffarpur, India; 2 Department of Microbiology College, Gouri Devi Institute of Medical Sciences & Hospital Durgapur, West Bengal, India; 3 Department of General Surgery, Sri Krishna Medical College Muzaffarpur, Bihar, India; 4 Department of Surgical Gastroenterology and Liver Transplant, Indira Gandhi Institute of Medical Sciences, Patna, Bihar, India; 5 Department of Surgery, Tata motors hospital Jamshedpur, India; 6 Department of Pathology, Nalanda Medical College & Hospital, Patna, Bihar India

**Keywords:** Minimally invasive surgery, patient awareness, attitudes, community health

## Abstract

Minimally invasive surgery (MIS) has brought significant advancements in surgical practices. However, patient awareness and attitudes towards MIS are not well-documented in community health settings like Muzaffarpur and Bihar. This study aims to evaluate the factors among patients to improve understanding and acceptance of MIS in these settings. The study involved 350 adult patients visiting the outpatient department. A structured questionnaire assessed participants' awareness, attitudes and perceptions of MIS. The study underscores the need for enhanced patient education and communication strategies regarding MIS, especially in community health settings. Tailored educational programs can potentially bridge the gap between advancements in surgical techniques and patient awareness, fostering informed healthcare decisions.

## Background:

Minimally invasive surgery (MIS) offers significant advantages over traditional surgery, including reduced postoperative discomfort, faster recovery and less scarring [1]. Despite these benefits, research on patient understanding and attitudes towards MIS is limited, especially in community health settings like Muzaffarpur, Bihar [2]. This study aims to address this gap by examining patient perceptions of MIS at Sri Krishna Medical College and Hospital, a key healthcare facility in the region. Patient knowledge and perceptions significantly influence healthcare decisions, particularly in areas with limited access to specialized medical services [3]. Understanding patient views on advanced surgical procedures like MIS is crucial in settings like Muzaffarpur, where healthcare resources may be scarce [4, 5]. The study highlights that ensuring equitable access to modern medical interventions, such as minimally invasive surgery (MIS), is a significant challenge in regions like Bihar, particularly due to socioeconomic disparities. The research underscores the necessity of tailored educational programs and communication strategies to address these inequities and improve patient awareness, as suggested by Malhotra and Do's work on health policy planning [6]. The insights from this research are aligned with Mohan and Kumar's findings, emphasizing that improved patient knowledge and acceptance of advanced medical interventions like MIS require a collaborative effort among educators, policymakers and healthcare professionals. This study's outcomes will assist these stakeholders in implementing effective strategies to bridge the awareness gap in community health settings [7]. By addressing the gap between advancements in surgical techniques and patient awareness in this context, our study aims to foster a healthcare system where patients are well-informed and empowered to make optimal health decisions [8].

## Methods and Materials:

## Study design and setting:

This cross-sectional survey was conducted at Sri Krishna Medical College and Hospital in Muzaffarpur, Bihar. The study assessed patient awareness and attitudes toward minimally invasive surgery (MIS) in this community health setting. The survey period extended from July to December 2023.

## Study population and sampling:

The study population consisted of adult patients (aged 18 years and above) visiting the hospital's outpatient department. We employed a stratified random sampling method to ensure representation across various demographic segments, including age, gender and socio-economic status. An estimated sample size of 350 participants was calculated to achieve a 95% confidence level with a 5% margin of error, considering the expected patient flow and diversity.

## Survey instrument:

A structured questionnaire was developed, including demographic questions and specific items related to MIS. The survey measured awareness (knowledge about MIS and its benefits), attitudes (preferences, perceived risks) and prior experiences with surgical procedures, if any. The content validity of the questionnaire was ensured through expert reviews by local healthcare professionals and its reliability was established through a pilot test with 30 participants.

## Data collection procedure:

Data were collected through face-to-face interviews conducted by a team of trained interviewers. Interviews were held in a private, quiet hospital area to ensure participant comfort and confidentiality. Informed consent was obtained from each participant before the survey. Participants were informed about the purpose of the study and anonymity was assured.

## Data analysis:

The collected data were entered into a computerized database and analyzed using SPSS software (version 26). Descriptive statistics were used to summarize demographic data and survey responses. Inferential statistics, such as Chi-square tests, were used to examine the relationships between demographic factors and attitudes toward MIS. A p-value of <0.05 was considered statistically significant for all analyses.

## Ethical considerations:

The study protocol was reviewed and approved by the Institutional Ethics Committee of Sri Krishna Medical College and Hospital. All procedures performed were in accordance with the ethical standards of the institutional research committee and with the 1964 Helsinki Declaration and its later amendments or comparable ethical standards.

## Results:

The study successfully surveyed 350 participants at Sri Krishna Medical College and Hospital. The results are presented in the following tables: Table 1 provides a breakdown of the study participants' demographics. It shows a nearly even gender distribution, with a slight majority of males (51.4%). The age distribution indicates a good representation across different age groups, with the largest group being 31-45 (35.7%). In terms of education, most participants have at least secondary education (35.7%), reflecting a relatively educated sample. The participants' socioeconomic status varied, with the largest group falling into the low-income category (42.9%). Table 2 highlights the varying levels of awareness about MIS among participants. 22.9% of the participants have a high awareness level, while the majority (42.9%) has a moderate awareness. A significant portion (34.3%) has low awareness, indicating a need for improved education and information dissemination about MIS. The attitudes towards MIS are generally positive. 57.1% of participants show a positive attitude towards preferring MIS, while 22.9% are neutral and 14.3% have a negative attitude. Similar trends are observed in the perceived safety of MIS and willingness to recommend MIS, suggesting overall positive perceptions but also indicating areas where attitudes could be improved. Most participants recognize the benefits of MIS. A significant majority identify reduced recovery time (71.4%) and less postoperative pain (65.7%) as major benefits. Over half of the participants acknowledge a lower risk of complications (51.4%) and a shorter hospital stay (57.1%). These findings underscore the importance of communicating these benefits to patients. Participants expressed several concerns regarding MIS (Table 3, Table 4, Table 5). The lack of information is the most common concern (42.9%), followed by fear of unknown complications (34.3%) and doubts about its effectiveness (28.6%). Accessibility and availability are also concerns for 22.9% of participants, highlighting areas where patient education and healthcare infrastructure need improvement. Table 6 shows the relationship between demographic factors and attitudes towards MIS. Both genders exhibit similar positive attitudes, with slightly more males showing a positive attitude. Younger participants (18-30 years) are more likely to have a positive attitude than older participants, suggesting generational differences in receptivity to MIS. Education level appears to correlate with a positive attitude, as those with higher education levels show more positivity towards MIS.

## Discussion:

This study at Sri Krishna Medical College and Hospital in Muzaffarpur, Bihar, provides crucial insights into patient awareness and attitudes toward Minimally Invasive Surgery (MIS) in a community health setting. The findings reveal moderate to low awareness of MIS among participants, consistent with other Indian studies, suggesting a nationwide variation in understanding modern medical procedures [9, 10]. Our study highlights MIS surgeons have limited awareness to the challenge of the pandemic for the patient care which is in contrast to the study of Balakrishna *et al.* [11] Similar to programs in states such as West Bengal and Karnataka, these results underscore the need for educational outreach [12, 13]. The positive perception of MIS aligns with India's growing preference for less invasive treatments [14]. Conversely, neutral and negative attitudes in other studies indicate that certain groups continue to hold misconceptions and concerns about new medical technologies [15]. Participants' perceptions of the benefits of MIS mirror findings from national patient surveys [7, 16]. The concerns related to MIS, particularly information gaps, are similar to those identified in healthcare settings in Tamil Nadu and Uttar Pradesh [17, 18]. The study corroborates findings from Rahimi *et al.*, showing that demographic factors such as age play a pivotal role in attitudes toward MIS. Younger individuals, particularly those aged 18-30, demonstrate a more positive attitude towards medical innovations, indicating generational differences in receptivity [19]. Education level significantly influences attitudes toward MIS, aligning with Dhagarra *et al.*'s observations that individuals with higher education are more likely to embrace advanced medical procedures. The correlation highlights the importance of educational interventions to improve awareness and acceptance [20]. Comparing our results with other Indian studies suggests that regional healthcare infrastructure, educational initiatives and cultural perceptions influence awareness and attitudes toward MIS, which vary across the country [10, 11].

## Limitations and future research:

The study's focus on a single institution in Bihar may limit its generalizability. Future research should include multiple healthcare settings across different Indian states for a more comprehensive understanding.

## Conclusion:

Our study highlights the necessity for tailored educational programs and communication strategies to enhance patient understanding and acceptance of MIS in India. Such efforts are crucial for ensuring equitable access to advanced medical interventions across diverse socio-economic and geographic landscapes.

## Figures and Tables

**Table 1:** Demographic characteristics of participants

**Variable**	**Number of Participants**	**Percentage (%)**
**Gender**		
Male	180	51.4
Female	170	48.6
Age Group		
18-30 years	100	28.6
31-45 years	125	35.7
46-60 years	85	24.3
Above 60 years	40	11.4
Education Level		
No formal education	50	14.3
Primary	75	21.4
Secondary	125	35.7
Higher	100	28.6
Socioeconomic Status		
Low	150	42.9
Middle	125	35.7
High	75	21.4

**Table 2:** Awareness of minimally invasive surgery

**Awareness Level**	**Number of Participants**	**Percentage (%)**
High	80	22.9
Moderate	150	42.9
Low	120	34.3

**Table 3:** Attitudes towards minimally invasive surgery

**Attitude Aspect**	**Positive**	**Neutral**	**Negative**
Preference for MIS	200	100	50
Perceived Safety of MIS	220	90	40
Willingness to Recommend MIS	230	80	40

**Table 4:** Perceived benefits of minimally invasive surgery

**Benefit**	**Number of Participants**	**Percentage (%)**
Reduced Recovery Time	250	71.4
Less Postoperative Pain	230	65.7
Lower Risk of Complications	180	51.4
Shorter Hospital Stay	200	57.1

**Table 5:** Concerns about minimally invasive surgery

**Concern**	**Number of Participants**	**Percentage (%)**
Lack of Information	150	42.9
Fear of Unknown Complications	120	34.3
Doubts About Effectiveness	100	28.6
Accessibility and Availability	80	22.9

**Table 6:** Correlation between Demographics and Attitudes towards MIS

**Concern**	**Number of Participants**	**Percentage (%)**
Lack of Information	150	42.9
Fear of Unknown Complications	120	34.3
Doubts About Effectiveness	100	28.6
Accessibility and Availability	80	22.9
